# Phase II study of the farnesyltransferase inhibitor R115777 in advanced melanoma (CALGB 500104)

**DOI:** 10.1186/1479-5876-10-246

**Published:** 2012-12-10

**Authors:** Thomas F Gajewski, April KS Salama, Donna Niedzwiecki, Jeffrey Johnson, Gerald Linette, Cynthia Bucher, Michelle A Blaskovich, Said M Sebti, Frank Haluska

**Affiliations:** 1 The University of Chicago, Section of Hematology/Oncology, 5841 S. Maryland Ave., MC2115, Chicago, IL, 60637, USA; 2 Duke University Medical Center, Section of Medical Oncology, Durham, NC, 27710, USA; 3 CALGB Statistical Center, Durham, NC, 27710, USA; 4 Washington University, St Louis, MO, USA; 5 Drug Discovery Department, Moffitt Cancer Center, Tampa, FL, 33612, USA; 6 ARIAD Pharmaceuticals, Inc., Cambridge, MA, 02139, USA

**Keywords:** Melanoma, Farnesyltransferase inhibitor, Tipifarnib, R11577, RAS, T cell activation

## Abstract

**Background:**

Multiple farnesylated proteins are involved in signal transduction in cancer. Farnesyltransferase inhibitors (FTIs) have been developed as a strategy to inhibit the function of these proteins. As FTIs inhibit proliferation of melanoma cell lines, we undertook a study to assess the impact of a FTI in advanced melanoma. As farnesylated proteins are also important for T cell activation, measurement of effects on T cell function was also pursued.

**Methods:**

A 3-stage trial design was developed with a maximum of 40 patients and early stopping if there were no responders in the first 14, or fewer than 2 responders in the first 28 patients. Eligibility included performance status of 0–1, no prior chemotherapy, at most 1 prior immunotherapy, no brain metastases, and presence of at least 2 cutaneous lesions amenable to biopsy. R115777 was administered twice per day for 21 days of a 28-day cycle. Patients were evaluated every 2 cycles by RECIST. Blood and tumor were analyzed pre-treatment and during week 7.

**Results:**

Fourteen patients were enrolled. Two patients had grade 3 toxicities, which included myelosuppression, nausea/vomiting, elevated BUN, and anorexia. There were no clinical responses. All patients analyzed showed potent inhibition of FT activity (85-98%) in tumor tissue; inhibition of phosphorylated ERK and Akt was also observed. T cells showed evidence of FT inhibition and diminished IFN-γ production.

**Conclusions:**

Despite potent target inhibition, R115777 showed no evidence of clinical activity in this cohort of melanoma patients. Inhibition of T cell function by FTIs has potential clinical implications.

Clinicaltrials.gov number NCT00060125

## Background

Metastatic melanoma is difficult to treat and it is only recently that therapy has been shown to have an impact on overall survival [1-3]. DTIC/dacarbazine has been shown in contemporary studies to provide tumor responses in less than 15% of patients, with a median response duration of 3–4 months [4,5]. Combination therapies may increase response rates, but without improvement in survival [6]. High dose interleukin-2 and ipilimumab benefit the minority of patients, albeit with a subset of patients experiencing durable responses [1,7,8]. Although many patients with BRAF-mutated melanoma initially respond to vemurafenib, the only other agent approved by the FDA for this disease, most will ultimately relapse [2]. Thus, while significant advances in both immune based and molecularly targeted therapies have been made, survival for many patients with metastatic melanoma remains poor. New therapies are still needed for this disease, and the testing of new agents is being driven by an increasing knowledge of melanoma biology.

The vast majority of melanomas have activating mutations in signaling proteins involved in the RAS pathway. Mutations in RAS occur in around 15% of melanomas [9,10]. In addition, frequent mutations in downstream RAS effectors have been reported, the most common of which is BRAF which has been reported to be mutated in approximately 50% of cases [11-13]. Mutated BRAF can be effectively targeted in patients with metastatic melanoma, with impressive response rates in early phase trials [14-16]. Recent data now demonstrates an improvement in overall survival in patients treated with selective BRAF inhibitors when compared to dacarbazine, although many patients ultimately relapse, further highlighting the importance of understanding the molecular pathogenesis of this disease [2,17]. Activation of the PI3 Kinase/Akt pathway has also been implicated in melanoma tumorigenesis, potentially through downregulated expression of the negative regulator PTEN [18-20]. Interestingly, even in melanoma cells having mutations in downstream effectors, constitutive RAS activation is nonetheless seen, likely through the activity of autocrine or paracrine growth factor secretion [21]. Transgenic mouse experiments have confirmed the important contribution of activated RAS-based signaling to melanomagenesis in vivo [22-24].

Targeted inhibition of RAS-based signaling has therefore received significant attention. While kinase inhibitors that interfere with the activity of the downstream molecules PI3 Kinase, RAF, and MEK are in various stages of development, it has been difficult to identify a pharmacologic strategy to inhibit RAS activity directly [25]. However, the fact that RAS must undergo a lipid post-translational modification for localization to membrane compartments where access to its effectors occurs generated an alternative strategy for inhibiting RAS function. The most important post-translational modification of RAS is farnesylation, which is catalyzed by the enzyme Farnesyltransferase (FT) [26]. FT inhibitors (FTIs) have been developed as a strategy to block this process, thereby decreasing RAS translocation to membranes and reducing its ability to mediate activation of downstream effectors [27]. Interestingly, despite the initial motivation of FTI development driven by an interest in inhibiting RAS, FTIs have subsequently been shown to have effects on numerous additional proteins involved in tumor survival and proliferation. These include other GTPases such as Rheb, Ral, RhoC and Rac1, as well as factors involved in regulated protein translation and angiogenesis [28,29]. Preclinical data have shown anti-proliferative activity that is independent of Ras mutation status, and mechanistic experiments have implicated alternative farnesylated targets as functionally relevant. Thus, FTIs may in fact target multiple signaling molecules that contribute to malignant transformation and are no longer viewed as pure RAS inhibitors [27]. Recently, there has also been evidence to suggest that FTIs may enhance the effectiveness of cytotoxic chemotherapy when used in combination, potentially expanding the role of these agents [30,31].

R115777 is an orally bioavailable methyl-quinolone, which has been shown to be a potent and selective inhibitor of FT in the nanomolar concentration range. Preclinical experiments demonstrated activity against melanoma tumor cell growth both in vitro and in vivo [32]. Phase I clinical trial testing identified a dose and schedule of R115777 of 300 mg PO BID, given for 21 days of a 28 day cycle which was sufficiently well tolerated for subsequent investigation [33,34]. The most common toxicities were myelosuppression, nausea, vomiting, and fatigue. Phase II studies have shown clinical activity as a single agent in patients with hematologic malignancies [35-39]. Together, these observations coalesced to motivate investigation of the FTI R115777 in patients with advanced melanoma. Inasmuch as there was limited experience in evaluating tumor tissue for effective biochemical target inhibition, an integral part of the current study involved obtaining sizable tumor tissue before and during R115777 administration to measure FT enzymatic activity directly and also to assess effects on specific signaling pathways ex vivo.

Many of the signaling pathways involved in melanomagenesis are also involved in T cell activation, including the RAS pathway [40]. We recently have shown that cytokine production and proliferation of T cells in response to T cell receptor (TCR) engagement is blocked in vitro by FTIs, suggesting that these compounds could theoretically inhibit T cell function in treated patients [41]. Given the importance of the immune system to participate in melanoma growth control, the effect of signal transduction inhibitors on lymphocyte function has become a critical parameter to consider in the cancer context [42]. This may be particularly relevant, given the recent data suggesting that selective inhibition of BRAF^V600E^ may increase T cell recognition of melanoma antigens in vitro [43]. Therefore, an additional goal of the current study was to assess whether T cell function in treated patients was affected ex vivo.

### Patients and methods

#### Study design

This was a multicenter phase II clinical trial of R115777 in patients with metastatic melanoma carried out by the CALGB melanoma working group. The primary objectives were to estimate the clinical response rate and to evaluate the toxicity of this agent in this patient population. The secondary objectives were to measure FT activity and effects on signaling events in tumor tissue, and to assess effects on T cell activation ex vivo from the peripheral blood.

#### Trial Conduct

CALGB developed and coordinated this trial. Institutional review board approval and patient informed consent were required at each participating center. Patient registration and data collection were managed by the CALGB Statistical Center. Data quality was ensured by careful review of data by CALGB Statistical Center staff and by the study chairperson. Statistical analyses were performed by CALGB statisticians.

As part of the quality assurance program of the CALGB, members of the Audit Committee visit all participating institutions at least once every three years to review source documents. The auditors verify compliance with federal regulations and protocol requirements, including those pertaining to eligibility, treatment, adverse events, tumor response, and outcome in a sample of protocols at each institution. Such on-site review of medical records was performed for a subgroup of 6 patients (43%) of the 14 patients under this study.

#### Patient selection

Patients must have had histologically confirmed melanoma with evidence for metastatic disease, either regional in-transit metastases not amenable to complete surgical resection or distant metastases. Treating physicians were required to discuss available standard therapies including DTIC and IL-2 prior to enrolling patients. Eligibility criteria included: the presence of at least two accessible lesions amenable to excisional biopsy for correlative assays; measurable disease in addition to the lesions planned for biopsy; absence of brain metastases; no allergies to azoles (e.g. ketoconazole); no more than one prior immunotherapy for metastatic disease; no prior chemotherapy for any stage of disease; ECOG performance status of at least 1; at least 18 years of age; non-pregnant and non-nursing; laboratory parameters within the following range: absolute neutrophil count ≥ 1500/μl; platelet count ≥ 100,000/μl; bilirubin ≤ 1.5 mg/dL; creatinine ≤ 2.0 mg/dL.

#### Treatment plan

R115777 was administered orally at a dose of 300 mg twice per day for 21 days of a 28-day cycle. Disease re-staging was performed every 2 cycles. Patients could remain on treatment until unacceptable toxicity or disease progression occurred. Prior to initiation of treatment, and again during week 7, an excisional biopsy was required to be performed for biologic correlates. At the same time points, heparinized blood was obtained for analysis of effects on T cells.

#### Evaluation of response and toxicities

Disease assessment was performed using RECIST criteria every two cycles. Toxicity evaluation was performed at least once per cycle. Dose reductions were allowed, with dose level −1 at 200 mg BID, dose level −2 at 100 mg BID, and dose level −3 being permanent discontinuation. For neurologic toxicity ≥ grade 2, drug was held until resolution to ≤ grade 1 and continued at a 1 level dose reduction. If the toxicity did not resolve within one week, then drug was permanently discontinued. For hematologic toxicities, if a grade 4 toxicity was observed then drug was held for up to 2 weeks. If resolution occurred to ≤ grade 1, then drug was resumed at a 1 level dose reduction. For other toxicities, if a grade 3 event was attributed to drug, then treatment was held up to 2 weeks. If toxicity resolved to ≤ grade 1, then drug was resumed at a 1 level dose reduction. If toxicity did not resolve within 2 weeks then drug was permanently discontinued. For any grade 4 non-hematologic toxicity attributed to drug, treatment was permanently discontinued.

#### Statistical considerations

A 3-stage design was used to allow for early termination if the drug appeared ineffective in this patient population. A maximum of 40 patients was targeted for enrollment, with the null hypothesis that the response rate (complete or partial response) is less than or equal to 0.05 versus the alternative hypothesis that the response rate is greater than or equal to 0.20. If no responses were observed in the first 14 patients then the trial would conclude accepting the null hypothesis. Otherwise, 14 patients would be enrolled in stage 2. If 2 or fewer total responses were observed, then the null hypothesis would be accepted. Else, the third stage would accrue to a total of 40 patients. If 4 or more responses were observed among 40 patients, then the drug would be considered efficacious. This procedure had a power of 0.91 and a significance level of 0.10. The probability of early termination was approximately 0.85 under the null hypothesis. For the correlative assays, descriptive statistics were proposed to describe changes in post-treatment versus pre-treatment specimens.

#### Tumor biopsies and assays for FT activity and RAS pathway signaling

Excisional biopsies were performed to obtain sufficient tissue for analysis and to minimize sampling error. Tissue was rapidly processed and stored until batched analysis. Proteins were extracted from snap-frozen tumor tissue using a tissue protein extraction reagent from Pierce (200 mg tumor/1 ml reagent). After homogenization at 4°C, the samples were spun at 13,000 x g and the supernatant found between the fatty top layer and the pellet was used for biochemical analysis. The FTase enzymatic assays as well as Western blots for protein level determination were carried out as described previously [35,44]. All analyses were performed in the laboratory of Dr. Said Sebti, at Moffitt Cancer Center.

#### Measurement of FTI action on T cells ex vivo

Peripheral blood mononuclear cells (PBMC) were separated from heparinized blood samples and stored as viable cells in freezing medium until batch analysis. Briefly, cells were thawed, cultured with the superantigen Staphylococcal enterotoxin A (SEA) or with Phorbol Myristate Acetate (PMA)/Ionomycin as a positive control, with or without the addition of R115777 *in vitro* as a comparison. After overnight culture, supernatants were analyzed for IFN-γ content by ELISA using antibody pairs from Pharmingen. Post-versus-pre-treatment samples were compared using a paired t-test. In parallel, cells were lysed and analyzed by Western blotting for the apparent molecular weight of the farnesylated protein HDJ-2 as described previously [45].

## Results

### Patient characteristics

Fourteen patients with metastatic melanoma were enrolled in this study between May 2003 and April 2005. The median age was 56 years (range: 36–89), and 9 (64%) were male. Five patients reported prior immunotherapy for metastatic disease, and 7 had an elevated LDH (greater than institutional ULN).

### Toxicity and clinical response

Treatment with R115777 was generally well tolerated. Only two patients showed grade 3 toxicities. One patient experienced grade 3 nausea and vomiting, which was associated with an increased serum BUN. A second patient experienced grade 3 myelosuppression and anorexia. These adverse events were readily reversible. Table 1.

**Table 1:** Toxicities associated with administration of R115777

	***Grade of Adverse Event***			
	**2**	**3**	**4**	**5**
	**n (%)**	**n (%)**	**n (%)**	**n (%)**
**Hematologic Adverse Events**				
Anemia	1 (8)	1 (8)	0 (0)	0 (0)
Leukopenia	2 (17)	0 (0)	0 (0)	0 (0)
Neutropenia	0 (0)	1 (8)	1 (8)	0 (0)
** Maximum Hematologic AEs**	1 (8)	1 (8)	1 (8)	0 (0)
**Non-Hematologic Adverse Events**				
Constitutional				
Fatigue	4 (33)	0 (0)	0 (0)	0 (0)
Gastrointestinal				
Anorexia	1 (8)	1 (8)	0 (0)	0 (0)
Dehydration	0 (0)	1 (8)	0 (0)	0 (0)
Diarrhea	1 (8)	0 (0)	0 (0)	0 (0)
Nausea	1 (8)	1 (8)	0 (0)	0 (0)
Vomiting	1 (8)	1 (8)	0 (0)	0 (0)
Metabolic				
Creatinine	0 (0)	1 (8)	0 (0)	0 (0)
Hyperglycemia	1 (8)	0 (0)	0 (0)	0 (0)
Neuropathy	1 (8)	0 (0)	0 (0)	0 (0)
Pain	1 (8)	0 (0)	0 (0)	0 (0)
Dyspnea	1 (8)	0 (0)	0 (0)	0 (0)
**Maximum Non-Hematologic AE**	5 (42)	2 (17)	0 (0)	0 (0)
**Overall Adverse Events**	5 (42)	2 (17)	1 (8)	0 (0)

Clinical response was assessed using RECIST criteria. There were no objective partial or complete responses observed in this cohort of 14 patients. Four patients exhibited stable disease and went on to a second course of therapy but progressed after an additional two cycles. All remaining patients progressed during the first cycle of treatment.

### Effects on farnesyltransferase (FT) enzymatic activity and selected signaling proteins in tumor tissue

**Figure 1:**
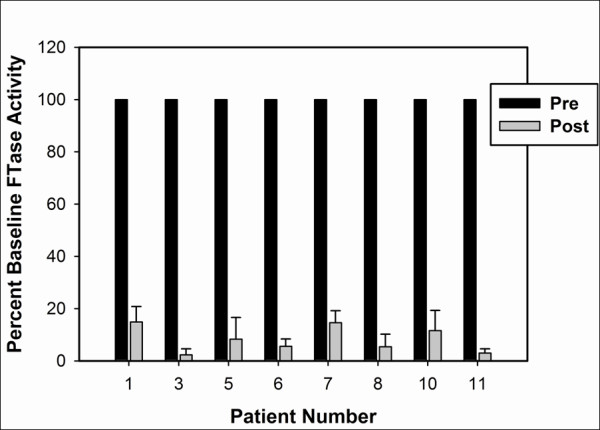
**Measurement of FT activity in tumor biopsies pre-treatment compared to on-therapy.** Excisional biopsies of cutaneous melanoma metastases obtained prior to therapy with R115777 and during week 7 of therapy were cryopreserved until analysis. FT activity was assessed as described in Materials and Methods. All post-treatment values were statistically significantly inhibited using an unpaired t-test (p<0.01). The assay was performed on single tumors in triplicate, and the error bars represent the variation in the assay for each sample.

Lack of clinical efficacy with an agent targeting a signaling pathway could be due to insufficient target inhibition, pathway modulation, or alternatively could be a reflection of tumor growth despite successful target blockade. In order to measure directly the biological effect of R115777 on its target FT, tumor biopsies obtained before and during week 7 of treatment were analyzed for FT enzymatic activity. Eight patients generated tumor tissue that contained sufficient quantity and quality of protein at both time points for analysis. As shown in Figure 1, FT enzymatic activity was suppressed by 85-98% in all tumor tissues analyzed comparing the week 7 to the pre-treatment time points. These results indicate that the target protein was inhibited very effectively in tumor tissue with the dose and schedule of R115777 used.

**Figure 2:**
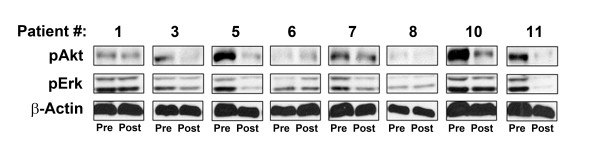
**Analysis of phospho-ERK and phospho-Akt in tumor biopsies pre-treatment compared to on-therapy.** Excisional biopsy material obtained prior to therapy with R115777 and during week 7 of therapy was analyzed by Western blot for the presence of phospho-Akt, phospho-Erk, and total β-Actin.

Although FT inhibition could result in multiple signaling proteins accumulating in a non-farnesylated form, if RAS itself was among the proteins affected, then inhibition of downstream effectors of RAS, such as ERK and Akt, might be observed. Indirect mechanisms to inhibit ERK and Akt activation also are conceivable. To test this notion, Western blot analysis was performed for phospho-ERK and phospho-Akt in the same tumor samples described above. Total β-actin was used as a loading control. As shown in Figure 2, constitutive phosphorylation of both ERK and Akt was detected at baseline in most of the samples analyzed. Interestingly, in several samples a marked decrease in detectable phospho-ERK and phospho-Akt was noted in the post-treatment samples (e.g. patients 3, 5, 10, and 11.). As none of these patients experienced tumor shrinkage, these results suggest that significant inhibition of measurable ERK and Akt activation can occur in melanoma metastases without a demonstrable clinical response.

### Effects on peripheral blood T cell function

**Figure 3:**
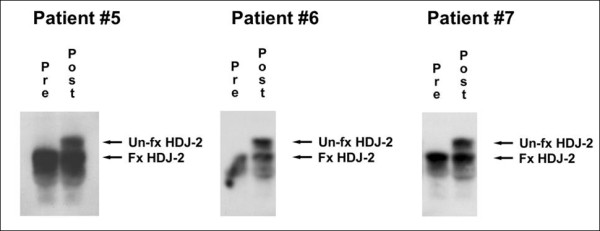
**HDJ-2 gel shift in peripheral blood lymphocytes analyzed ex vivo.** PBMCs obtained pre-treatment and during week 7 of therapy were analyzed by Western blot for the apparent molecular weight of the farnesylated protein HDJ-2. The appearance of a higher molecular weight band is indicative of a non-farnesylated state.

**Figure 4:**
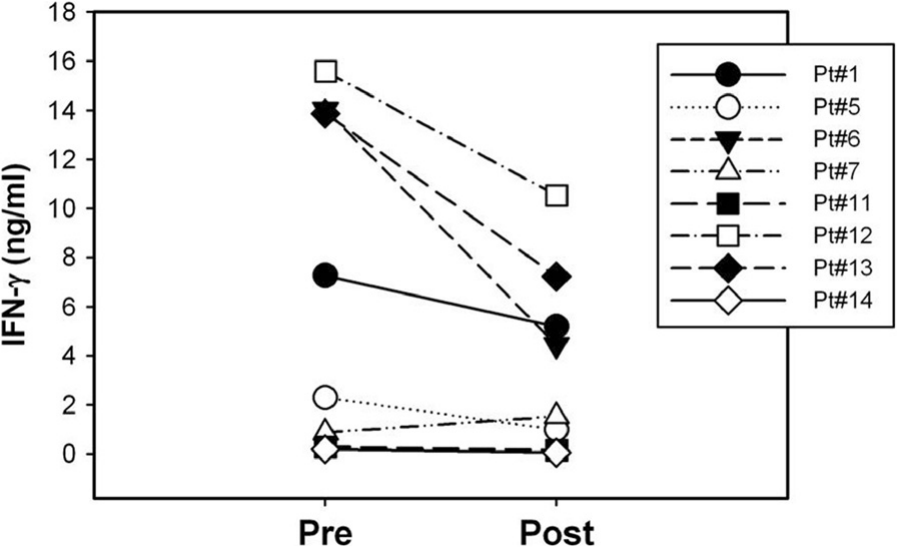
**IFŇγ production by peripheral blood lymphocytes analyzed ex vivo.** PBMC obtained pre-treatment and during week 7 of therapy were analyzed for T cell function by stimulating with the superantigen SEA and measuring IFN-γ production by ELISA. The post-versus pre-treatment values for the group of samples were statistically different as measured using a paired t-test (p<0.05).

The host immune response is thought to play a significant role in controlling metastatic melanoma, and this tumor type can be quite responsive to immunotherapeutic interventions [46]. We recently reported that FTIs can inhibit T cell activation through the T cell receptor (TCR) complex [41]. Therefore, it was of interest to determine whether there was evidence of suppression of T cell function from the peripheral blood cells of patients treated with R115777. We previously had reported that Western blot analysis of HDJ-2 could be used as a surrogate for farnesylation status in hematopoietic cells [41]. We therefore applied that assay to peripheral blood T cells. As shown in Figure 3 for three representative patients, accumulation of non-farnesylated HDJ-2 was easily detected in T cells at the week 7 time point. These results indicate that farnesylation was inhibited in peripheral blood T cells as it had been in the tumor tissue. To gauge whether T cell function could be affected by this inhibition of protein farnesylation, IFN-γ production was assessed on T cells stimulated ex vivo with the polyclonal stimulus, SEA. The combined data from all available patients are shown in Figure 4. Significant inhibition of IFN-γ production was observed in the week 7 samples compared to pre-treatment specimens. These results suggest that R115777 is capable of inhibiting T cell activation in humans in vivo.

## Conclusions

Potent anti-tumor effects of FTIs on melanoma cells in vitro motivated clinical exploration of R115777 in patients with advanced melanoma. Although the drug was well tolerated, and potent inhibition of FT in tumor tissue was documented, no clinical activity was observed in this cohort of patients. While it is conceivable that inhibition of FT activity by 85-98% is not enough to achieve an anti-tumor effect and that complete target inhibition may be required, these results nonetheless suggest that inhibition of FT alone will not be sufficient for clinical activity in melanoma. One caveat of this interpretation is that, while pre-treatment samples were analyzed by pathology to confirm the presence of melanoma, given the large amount of tissue needed to perform the correlative analyses, post-treatment samples were not routinely assessed for viable tumor. It is therefore technically possible that the decrease in FT activity seen in the post-treatment samples could be due to inadequate tumor in the sampled tissue, as a result of either necrosis or contamination with adjacent normal tissue. Given that marked FT inhibition was seen in multiple clinically evident lesions post therapy, and that no clinical responses were observed, it is most likely that these results reflect true target inhibition.

A recent clinical trial in patients with acute myelogenous leukemia has shown that patients whose tumor cells have a high ratio of expression of two genes, RASGRP1 and APTX, are more likely to respond to R115777 [47]. Therefore, in future trials it might be of interest to determine if this gene expression ratio is also indicative of the dependence of melanoma tumors on farnesylation. Therefore, the selection of patients whose melanoma tumors express such a high ratio may have a greater likelihood of clinical responses. Understanding the mutation status of RAS, BRAF and PI3K may also be informative for predicting tumor sensitivity resistance and would be important for future work.

The mechanism of anti-tumor activity of FTIs when they are effective is incompletely understood, and the majority of FTI trials have failed to demonstrate meaningful clinical activity, despite confirmation that FTase or another intended target was inhibited. Multiple mechanisms of resistance and escape have been proposed. It is possible, for example, that NRAS escapes the dependence on farnesylation and alternatively undergoes prenylation by geranylgeranyltransferase 1 (GGT 1). Furthermore, a better understanding of the clinically relevant FTI substrates is clearly needed, enabling better patient selection. Multiple proteins undergo prenylation, and it is likely that many are yet to be identified. RAS family proteins represent only a subset of molecules that undergo post-translational modification through farnesylation, and several alternative targets have been proposed that may be the most relevant for inhibition of tumor cell growth [27]. Interestingly, using normal murine and human T cells as a model system, we have observed that FTIs inhibited TCR-dependent cytokine production under conditions in which RAS pathway signaling was unaffected. Rather, in that system, inhibition of cytokine production appeared to occur at the post-transcriptional level and was associated with inhibition of p70S6 Kinase activation [41]. Rheb is a candidate farnesylated protein that activates the p70S6 Kinase pathway [48]. In vitro data suggest that the FTI lonafarnib may enhance the effects of the RAF inhibitor sorafenib via inhibition of mTOR signaling by blocking Rheb farnesylation [49]. Subsequent studies have shown that inhibition of mTOR signaling with lonafarnib augments sorafenib-induced apoptosis in melanoma cell lines. Interestingly, this effect seemed to be independent of BRAF or NRAS mutation status [50]. Thus, while these agents were initially developed as RAS inhibitors, our collective data suggest that the effects of FTIs likely affect multiple signaling pathways.

Of note, a randomized phase II trial comparing sorafenib in combination with either the mTOR inhibitor temsirolimus or R115777 in an unselected patient population failed to demonstrate meaningful clinical activity [51]. It is now known, however, that sorafenib is inactive in patients with BRAF-mutated melanoma, and the role of combination therapy with the newer selective BRAF inhibitors in patients whose tumors carry the BRAF^V600E^ mutation is unknown. However, the knowledge that the effect of lonafarnib appeared to be independent of mutational status provides theoretical basis for molecularly targeted therapy in patients whose tumors are wild-type for BRAF, a group who currently has no such option available. Additionally, recent data suggests that selective BRAFV600 inhibition does not impair the immune response [52]. Taken together, these data suggest that combination therapy of an FTI with a more selective BRAF inhibitor, with or without immunotherapy, may represent potential treatment strategies in the future for appropriately selected patients.

Several patients on this study demonstrated inhibition of ERK and Akt phosphorylation in tumor tissue following treatment with R115777, yet they did not have a clinical response. It is important to emphasize that reduced phospho-ERK and phosho-Akt does not prove that Ras proteins themselves were inhibited, as indirect effects are also conceivable. While the amount of tissue available limited the number of signaling proteins that could be analyzed after the FT assay was performed using most of the sample, this observation suggests either that more complete blockade of these pathways is necessary in order to have tumor regression, or that salvage mechanisms can arise that enable tumor growth despite inhibition of these pathways. Recent experience with BRAF inhibitors has suggested that a very high level of pathway inhibition (>90%) is necessary in order to achieve clinical tumor shrinkage [14]. One hypothetical salvage mechanism is through regulated expression of MAP Kinase phosphatases, which might be highly expressed in tumor cells that have constitutive ERK activation, but may decrease in expression when the ERK pathway is partially inhibited, thus resulting in little change in the final output of ERK phosphorylation of target genes. These and other potential mechanisms of resistance will be of interest to pursue in future studies of targeted inhibitors in melanoma.

## Competing interest

The authors declare that they have no competing interests.

## Authors’ contributions

TG conceived the study, participated in its design and coordination, and helped to draft the manuscript. AS interpreted data and helped draft the manuscript. DN and JJ participated in the design of the study and performed the statistical analysis. CB and MB performed the laboratory experiments. GL and FH helped conceive the study and participated in its design and coordination. SS participated in the study design, and supervised and interpreted the correlative analyses. All authors read and approved the final manuscript.
